# Biochemical and genetic predictors and correlates of response to lamotrigine and folic acid in bipolar depression: Analysis of the CEQUEL clinical trial

**DOI:** 10.1111/bdi.12531

**Published:** 2017-08-20

**Authors:** EM Tunbridge, MJ Attenburrow, A Gardiner, JM Rendell, C Hinds, GM Goodwin, PJ Harrison, JR Geddes

**Affiliations:** ^1^ Department of Psychiatry University of Oxford Oxford UK; ^2^ Oxford Health NHS Foundation Trust Oxford UK; ^3^ National Institute for Health Research (NIHR) Oxford Health Biomedical Research Centre Oxford UK; ^4^ NIHR CLAHRC Oxford Oxford Health NHS Foundation Trust Oxford UK; ^5^ Department of Primary Care University of Oxford Oxford UK; ^6^ Oxford University Big Data Institute University of Oxford Oxford UK

**Keywords:** bipolar disorder, catechol‐O‐methyltransferase, folate, folate hydrolase, glutathione, homocysteine, methylene tetrahydrofolate reductase

## Abstract

**Objectives:**

CEQUEL (Comparative Evaluation of QUEtiapine plus Lamotrigine combination versus quetiapine monotherapy [and folic acid versus placebo] in bipolar depression) was a double‐blind, randomized, placebo‐controlled, parallel group, 2×2 factorial trial that examined the effect of adding lamotrigine and/or folic acid (FA) to quetiapine in bipolar depression. Lamotrigine improved depression, but its effectiveness was reduced by FA. We investigated the baseline predictors and correlates of clinical response, and the possible basis of the interaction.

**Methods:**

The main outcome was change in depressive symptoms at 12 weeks, measured using the Quick Inventory for Depressive Symptoms—self report version 16 (QIDS‐SR16). We examined the relationship between symptoms and lamotrigine levels, and biochemical measures of one‐carbon metabolism and functional polymorphisms in catechol‐O‐methyltransferase (COMT), methylene tetrahydrofolate reductase (MTHFR) and folate hydrolase 1 (FOLH1).

**Results:**

Lamotrigine levels were unaffected by FA and did not differ between those participants who achieved remission and those with persisting symptoms. When participants with subtherapeutic serum levels were excluded, there was a main effect of lamotrigine on the main outcome, although this remained limited to those randomized to FA placebo. None of the biochemical measures correlated with clinical outcome. The negative impact of FA on lamotrigine response was limited to COMT Met carriers. FOLH1 and MTHFR had no effect.

**Conclusions:**

Our results clarify that FA's inhibition of lamotrigine's efficacy is not a pharmacokinetic effect, and that low serum lamotrigine levels contributed to lamotrigine's lack of a main effect at 12 weeks. We were unable to explain the lamotrigine−FA interaction, but our finding that it is modulated by the COMT genotype provides a starting point for follow‐on neurobiological investigations. More broadly, our results highlight the value of including biochemical and genetic indices in randomized clinical trials.

## 1 INTRODUCTION

Depression rather than mania accounts for the majority of the burden of increased mortality and long‐term disability in bipolar disorder.^1^ Treatment of bipolar depression remains a major clinical challenge with few treatment trials, particularly beyond the acute phase of illness, nor have many examined combination therapies, which are the norm in clinical practice.^2^, ^3^

We recently reported the results of the CEQUEL trial (Comparative Evaluation of QUEtiapine plus Lamotrigine combination versus quetiapine monotherapy [and folic acid versus placebo] in bipolar depression).^4^ CEQUEL was a multi‐centre, double‐blind, randomized clinical trial, with a 2×2 factorial design. It examined the efficacy of adding lamotrigine and/or folic acid (FA) to quetiapine monotherapy for depressive symptoms in participants with bipolar disorder. Participants were followed up at 12, 22 and 52 weeks, with changes in self‐reported depressive symptoms at 12 weeks as the prespecified outcome. The rationale for CEQUEL was that adding lamotrigine, which has a lengthy (6‐week) titration period, to quetiapine, which is relatively quick‐acting and is thought to act via different pharmacological mechanisms, might lead to synergistic long‐term outcomes.^4^ Similarly, FA, which is widely used as an over‐the‐counter vitamin supplement, has shown some clinical benefit in major depression,^5^ consistent with reports of a cerebral folate deficiency in patients with treatment‐refractory depression.^6^ Thus, we hypothesized that adding lamotrigine to quetiapine would be superior to quetiapine monotherapy, and that FA might be of additional benefit, independently of lamotrigine. The findings of CEQUEL were intriguing: instead of a clear benefit of lamotrigine over placebo at 12 weeks (*P*=.066), there was an interaction between FA and lamotrigine (*P*=.028). Specifically, FA appeared to block the therapeutic effect of lamotrigine at 12 weeks, although this interaction disappeared at later time points and lamotrigine was superior to placebo at 52 weeks.^4^

**Figure 1:**
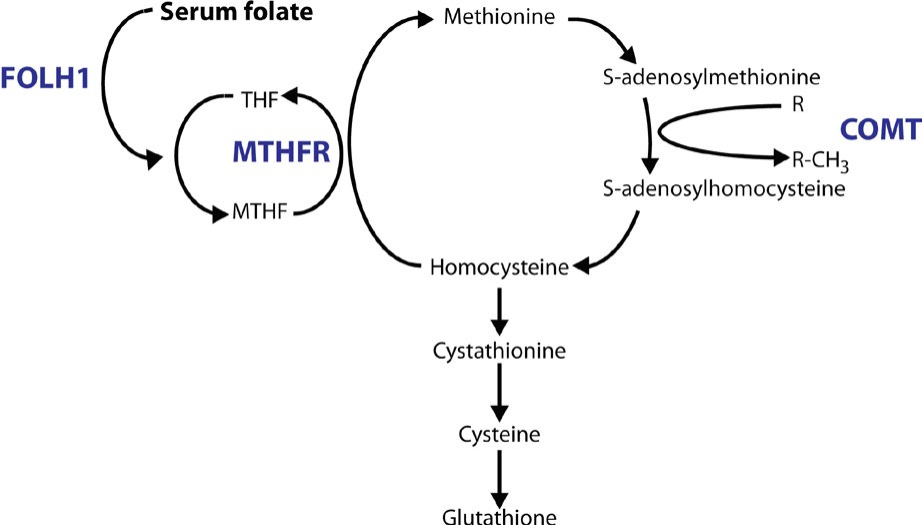
The one‐carbon cycle. The actions of folate hydrolase 1 (FOLH1), methylene tetrahydrofolate reductase (MTHFR) and catechol‐O‐methyltransferase (COMT) are highlighted in blue. Note that the activity of all S‐adenosylmethionine (SAM)‐dependent methyltransferases results in SAM's conversion to S‐adenosylhomocysteine (indicated by the conversion of “R” to “R‐CH_3_”). THF, tetrahydrofolate; MTHF, 5,10‐methylene tetrahydrofolate

FA's actions are generally ascribed to its effects on one‐carbon metabolism. FA is reduced to folates, which are essential cofactors and co‐substrates in this pathway (Figure 1). There are several single nucleotide polymorphisms which influence one‐carbon metabolism (Figure 1). The folate hydrolase 1 (FOLH1) C^484^T (rs202676) polymorphism may influence the binding potential of FOLH1.^7^ Dietary folates (but, notably, not FA) must be hydrolyzed by FOLH1 before they can be absorbed.^8^ Despite not directly influencing FA uptake, the FOLH1 C^484^T genotype has been implicated in the beneficial effect of FA on negative symptoms in patients with schizophrenia (Roffman et al., 2013). The methylene tetrahydrofolate reductase (MTHFR) C^677^T (rs1801133) polymorphism alters the activity of the encoded MTHFR (the T allele results in an enzyme with lower activity than that encoded by the wild‐type C allele), which is a key regulator of the folate cycle.^9^ Similarly, the catechol‐O‐methyltransferase (COMT) Val^158^Met (rs4680) polymorphism affects COMT's activity (the enzyme encoded by the ancestral Val allele has higher activity than that encoded by the Met allele).^10^ Because COMT converts its methyl donor into a homocysteine precursor, its activity indirectly affects one‐carbon metabolism (Figure 1). Notably, we showed interactive effects of these MTHFR and COMT polymorphisms on total homocysteine levels, such that levels were elevated in individuals carrying both MTHFR TT and COMT Val genotypes compared with other genotype groups, suggesting that the effects of these functional polymorphisms may be synergistic.^11^

In contrast, lamotrigine has multiple pharmacological actions and, although its anticonvulsant actions may result from its blockade of voltage‐gated sodium channels, it is unclear which of these are relevant for its efficacy in bipolar disorder.^12^ Interestingly, cellular and animal models suggest that lamotrigine has neuroprotective effects, mediated by reductions in oxidative stress.^13^, ^14^, ^15^ These findings are notable given evidence for increased oxidative stress in patients with bipolar disorder.^16^, ^17^

Given that the lamotrigine−FA interaction was unexpected, this aspect of the results of CEQUEL should be treated with caution. However, if correct, it would be of clinical significance given the widespread use of FA as a dietary supplement, particularly by women in the periconceptual and prenatal period, and its increasingly widespread fortification in flour and other grains around the world.

Here we present secondary analyses of the CEQUEL data, conducted with two goals in mind: first, to identify potential baseline predictors and correlates of the response to lamotrigine; second, to investigate the possible mechanistic basis of the observed lamotrigine−FA interaction. To address these issues, we conducted protocol‐defined analyses of biochemical and genetic measures, particularly related to one‐carbon metabolism, as well as exploratory assessments of lamotrigine and folate levels in the blood.

## 2 METHODS

### 2.1 Study design and participants

CEQUEL was a double‐blind, randomized, placebo‐controlled, parallel group, 2×2 factorial trial that was conducted across 27 UK sites. CEQUEL is described in detail in our earlier publication^4^ and the trial protocol is available from JRG. Briefly, following a 7−14‐day run‐in on quetiapine, patients with bipolar disorder type I or II (according to DSM‐IV^18^ criteria based on clinician interview) were randomly assigned to added lamotrigine or placebo and, separately, to FA or placebo. Lamotrigine was commenced at 25 mg daily and increased gradually to 200 mg (100 mg/day with concurrent valproate and 400 mg/day with concurrent combined oral contraceptives). Participants who were not already taking FA, and who had no contraindications to it, were separately randomized to FA (500 μg/day, a dose close to that of many over‐the‐counter supplements) or placebo. Participants were followed up at 12, 22 and 52 weeks; the main outcome was the change in depressive symptoms at 12 weeks, measured using the Quick Inventory for Depressive Symptoms—self report version 16 (QIDS‐SR16). All data were obtained between 21 October 2008 and 27 April 2013.

### 2.2 Trial protocol and ethics

Written informed consent was obtained from every patient. The study was approved by each site. CEQUEL was registered with EUdraCT (number 2007‐004513‐33; https://eudract.ema.europa.eu/) and approved by the Research Ethics Committee (REC 08/H0605/39), with clinical trial authorization (20584/0234/001‐0001 and ISRCTN17054996; http://www.isrctn.com/ISRCTN17054996). A number of protocol changes were made during the trial, all of which were approved by Oxfordshire REC B, as detailed previously.^4^ Most relevant here, Version 06 (May, 2013) included investigation of the effect of the folate hydrolase polymorphism on FA. The measurement of serum lamotrigine levels was approved by the University of Oxford's Central University Research Ethics Committee (approval number R05736/RE001).

### 2.3 Biochemical measures and genotyping

Plasma concentrations of folate, vitamin B_12_, total transcobalamin, holo‐transcobalamin and total homocysteine were determined as described previously^19^ in individuals who provided samples for biochemical analysis. Total homocysteine was not determined if the plasma sample was processed outside of 48 hours post‐collection; therefore, this information is missing for five individuals (see below for group allocations) for whom other biochemical measures were available. Genomic DNA was extracted from whole blood using the QIAamp Blood Midi kit (Qiagen, Southampton, UK). The COMT Val^158^Met (rs4680), MTHFR C^677^T (rs1801133) and FOLH1 C^484^T (rs202676) polymorphisms were genotyped using Taqman^™^ SNP Genotyping assays (COMT: C_25746809_50; MTHFR: C_1202883_20; FOLH1: custom assay ID# AH396WC; Thermo Fisher Scientific, Waltham, MA, USA) using an ABI Prism 7900HT thermal cycler (Thermo Fisher Scientific).

To investigate the possibility of a pharmacokinetic interaction between lamotrigine and FA, serum lamotrigine levels were determined in participants who: were randomized to lamotrigine; consented to the storage and future use of blood samples; continued on trial medication up to the 12‐week assessment; and provided a blood sample. Levels were thus estimated in 43 participants (n=20 allocated lamotrigine and FA [“FA positive”]; n=21 allocated lamotrigine and FA placebo; n=2 allocated lamotrigine but excluded from the FA arm and not taking FA at screening). For analysis purposes, those not randomized to FA were pooled with the FA placebo group (“FA negative”). Lamotrigine was assayed by liquid chromatography/mass spectrometry (LC/MS) by the Therapeutic Drug Monitoring Unit, Chalfont Centre for Epilepsy (Chalfont St Peter, UK). For the purposes of analysis, undetectable lamotrigine levels were coded as 0 mg/L and those with levels determined as <1 mg/L were coded as 0.5 mg/L. The therapeutic reference range used by the Unit for treatment of epilepsy is 3‐14 mg/L.

### 2.4 Data analysis

Statistical analyses were conducted in IBM SPSS Statistics version 24 (IBM, Armonk, NY, US). With the exception of defining “remission” and “non‐remission” groups, described below, the only clinical variable examined was the CEQUEL primary outcome: the change in QIDS‐SR16 score from baseline to 12 weeks (with baseline QIDS score included as a covariate). With the exception of the comparison of serum lamotrigine levels, detailed further below, analyses were limited to individuals randomized to both lamotrigine and FA. Biochemical measures were highly skewed and so were log_10_‐transformed (with a constant added prior to log transformation to ensure all values were positive). Genotyping results were pooled into two groups to increase statistical power. Specifically, COMT Val homozygotes were compared to Met carriers, and MTHFR C carriers to T homozygotes, as in our earlier study.^11^ For FOLH1, C carriers were compared to TT homozygotes, since C is the minor allele. Effects of COMT and MTHFR were examined within the same analyses, given that we have previously shown interactive effects between them on one‐carbon metabolism. FOLH1 was examined in separate analyses, since there was no a priori reason to anticipate a direct interaction with either COMT or MTHFR.

Lamotrigine levels were compared between FA positive (n=20) and FA negative (n=23) groups, and between those in remission from depression at 12 weeks (n=13; defined as 12‐week QIDS‐SR16 score ≤5) and a non‐remission group (n=30; defined as 12‐week QIDS‐SR16 score ≥6), using one‐way analysis of variance (ANOVA). The effects of genotype(s) and/or treatment (lamotrigine vs lamotrigine placebo; FA vs FA placebo) were examined using ANOVA in those randomized to FA (placebo−placebo, n=28; placebo−FA, n=34; lamotrigine−placebo, n=35; lamotrigine−FA, n=33), with least significant difference (LSD) post hoc tests used to explore significant interactions and planned comparisons. Univariate ANOVAs were used where the dependent variable was the primary clinical outcome. Baseline biochemical measures were compared between groups using one‐way ANOVA. To explore the effect of drug treatment and genotype measures on biochemical measures, repeated‐measures ANOVAs (with time—baseline vs 12 weeks—as the repeat factor) were used, since change scores remained non‐normally distributed even after log transformation (placebo−placebo, n=12; placebo−FA, n=19 [17 for total homocysteine]; lamotrigine−placebo, n=20 [19 for total homocysteine]; lamotrigine−FA, n=24 [22 for total homocysteine]). Additional univariate ANOVAs were used to explore the effect of genotype on baseline biochemical measures, since not all participants provided both baseline and 12‐week samples. Investigations of the effect of genotype on biochemical measures were conducted in a hypothesis‐driven manner. Thus, only FOLH1 genotype was included in the analysis of folate levels, only COMT was examined in the case of homocysteine (there were too few MTHFR TT homozygotes in the subset of individuals in whom biochemical measures were available to permit meaningful comparisons; Table 1), and none of the genotype measures were included in investigations of the B12 parameters.

**Table 1:** Baseline and 12‐week biochemical measures in different randomization groups

		Placebo−placebo	Placebo−lamotrigine	FA−placebo	Lamotrigine−FA	
N (baseline/12 weeks)		25/12	30/20	31/19	30/24	
Folate	Baseline	24.9±6.6 (29.4±5.9)	18.4±2.6 (18.8±4.5)	12.3±1.2 (11.3±4.7)	17.2±2.6 (18.3±4.2)	*F*_3,115_=1.6; *P*=.19
	12 weeks	15.2±5.6	16.6±4.3	38.4±4.5	45.3±4.0	
Homocysteine^a^	Baseline	13.7±2.1 (11.5±1.3)	11.2±0.6 (11.0±1.0)	14.7±1.2^b^ (15.9±1.1)	11.4±0.7 (11.5±0.9)	*F*_3,113_=2.3; *P*=.083
	12 weeks	9.3±1.1	10.1±0.9	12.0±0.9	8.6±0.8	
Vitamin B_12_	Baseline	338.9±26.6 (310.4±96.6)	416.3±92.6 (470.9±74.9)	277.3±16.0 (283.6±76.8)	352.9±25.3 (343.3±68.3)	*F*_3,115_=1.5; *P*=.22
	12 weeks	269.4±48.0	338.7±37.2	287.8±38.1	314.3±33.9	
Holo‐transcobalamin	Baseline	58.3±5.4 (54.7±11.8)	64.7±9.2 (69.3±9.2)	48.4±3.3 (45.7±9.6)	63.9±6.9 (65.5±8.4)	*F*_3,115_=0.8; *P*=.49
	12 weeks	51.8±10.5	68.7±8.1	47.6±8.6	57.1±7.4	
Total transcobalamin	Baseline	877.6±51.2 (905.1±68.6)	767.8±34.5 (743.0±53.1)	8626±37.6 (854.7±54.5)	851.3±40.8 (901.5±48.5)	*F*_3,115_=1.5; *P*=.21
	12 weeks	847.7±53.7	761.7±43.6	831.2±43.0	866.3±38.0	

Values in parentheses are baseline values for the subset of cases who also had 12‐week data and therefore included in the main analyses. Data are presented as mean ± standard error of the mean.

a Homocysteine data are missing for two individuals in the folic acid (FA) placebo group.

b Differs from placebo−lamotrigine (*P*=.023) and FA−lamotrigine (*P*=.031) groups.

The relationship between lamotrigine levels and clinical outcome was explored using partial correlation, controlling for FA group. Spearman's correlations were used to investigate the relationship between biochemical variables (baseline and 12‐week change scores) and clinical outcome.

## 3 RESULTS

### 3.1 The effect of lamotrigine may be limited by low serum levels in some individuals

**Figure 2:**
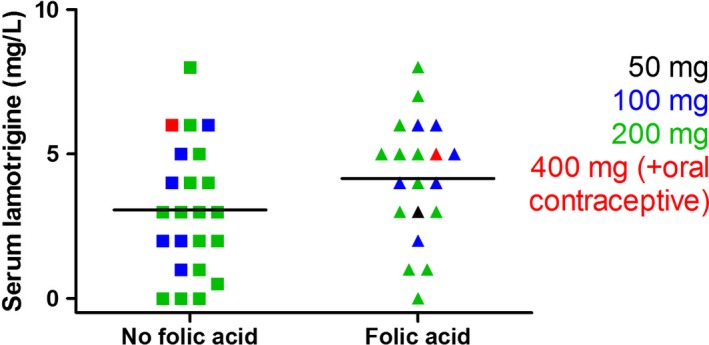
Serum lamotrigine levels at 12 weeks in CEQUEL (Comparative Evaluation of QUEtiapine plus Lamotrigine combination versus quetiapine monotherapy [and folic acid versus placebo] in bipolar depression) participants did not differ between folic acid (FA)‐positive (n=20) and FA‐negative (n=23) groups. Different coloured symbols indicate the dose of lamotrigine given, as shown in the key. A number of individuals had levels below the 3‐14 mg/L therapeutic range

We compared lamotrigine levels between FA‐positive and FA‐negative individuals to investigate whether the lamotrigine×FA interaction observed in the primary analysis might result from a pharmacokinetic effect. However, lamotrigine levels at 12 weeks did not differ between the FA‐positive and FA‐negative groups (*F*_1,42_=2.7; *P*=.108; Figure 2A), arguing against this hypothesis. The mean lamotrigine level was 3.6 mg/L (95% confidence interval [CI]: 2.9‐4.2 mg/L).

Notably, a number of individuals assigned to lamotrigine had low or undetectable serum lamotrigine levels (Figure 2A). Therefore, we examined the effect of lamotrigine and FA randomization excluding the 14 subjects with subtherapeutic lamotrigine levels (<3 mg/L)^20^ (n=10 randomized to FA placebo and n=4 randomized to FA) to investigate whether low lamotrigine levels in some individuals might contribute to the lack of a main effect of lamotrigine in the primary analysis. After excluding these individuals, and in contrast to the primary analysis,^4^ there was a main effect of lamotrigine (*F*_1,110_=5.3; *P*=.023). There was no main effect of FA (*F*_1,110_=0.01; *P*=.90), nor a lamotrigine−FA interaction (*F*_1,110_=1.8; *P*=.183). However, planned post hoc comparisons revealed that, as in our primary analysis, the beneficial effect of lamotrigine was seen in those randomized to FA placebo (*F*_1,110_=6.1; *P*=.015) and not in those randomized to FA (*F*_1,110_=0.5; *P*=.48).

We investigated whether there was a relationship between serum lamotrigine levels and clinical improvement. However, we found no correlation between serum lamotrigine and the clinical improvement at 12 weeks (partial correlation coefficient [controlling for FA group]=−0.08; *P*=.62). The measure of remission from depression was sensitive to lamotrigine treatment: 22 of 68 participants assigned to lamotrigine, vs only nine of 62 participants assigned to lamotrigine placebo (χ^2^=5.9; *P*=.017) had a QIDS‐SR16 score of ≤5 at 12 weeks. However, we observed no difference in lamotrigine levels between those who were in remission from depression [mean ± standard deviation (SD) 3.5±0.4 mg/L] and those who were not (3.8±0.5 mg/L; *F*_1,41_=0.2; *P*=.64).

### 3.2 The effect of FA randomization is influenced by COMT genotype

We investigated whether functional polymorphisms in genes related to one‐carbon metabolism might influence clinical response, given that FA's effects are generally ascribed to its modulation of this biochemical pathway. All polymorphisms were in Hardy−Weinberg equilibrium (COMT: χ^2^=1.122; *P*=.290; MTHFR: χ^2^=0.018; *P*=.892; FOLH1: χ^2^=0.170; *P*=.680).

**Figure 3:**
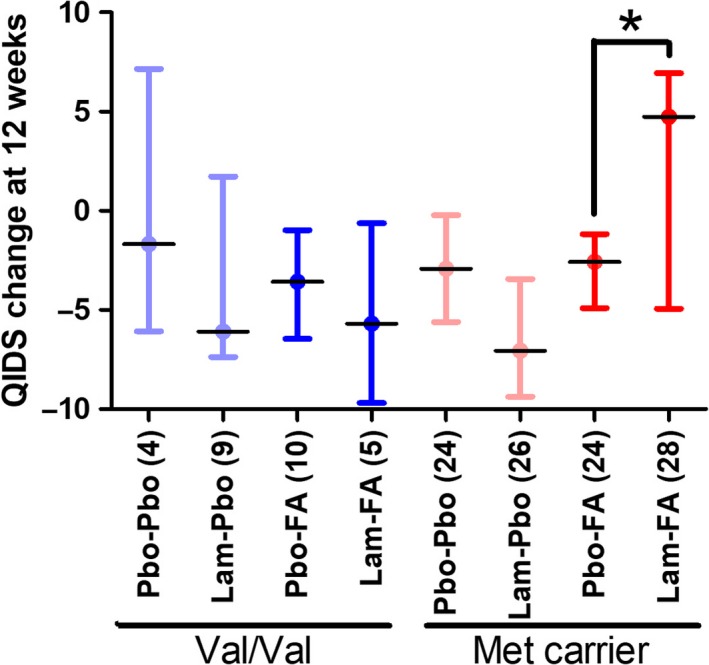
The effect of folic acid (FA) randomization on the primary clinical outcome depends on the catechol‐O‐methyltransferase (COMT) genotype. There was an interaction between COMT and FA, such that Val/Val homozygotes (FA placebo, n=13; FA, n=15) showed a greater improvement than Met carriers (FA placebo, n=43; FA, n=49). Planned comparisons indicated that the negative effect of FA on lamotrigine response was limited to Met carriers, since there was an effect of FA randomization in the Met carrier group randomized to lamotrigine (indicated with an asterisk) that was absent from all other groups (numbers in parentheses on the *x*‐axis indicate subgroup numbers). Lam, lamotrigine; Pbo, placebo; QIDS, Quick Inventory for Depressive Symptoms

The clinical effect of FA was influenced by COMT genotype (Figure 3). ANOVA revealed an interaction between COMT and FA (*F*_1,114_=6.4; *P*=.013; main effect of COMT: *F*_1,114_=3.3; *P*=.07). The interaction was due to a negative impact of FA in COMT Met carriers (*P*=.003) but not in COMT Val/Val homozygotes (*P*=.70). There was no COMT−FA−lamotrigine interaction (*F*_1,114_=0.12; *P*=.73). However, given the presence of the anticipated interaction between FA and lamotrigine (*F*_1,114_=6.5; *P*=.012) and the explicit aim of these analyses to explore this interaction, we performed exploratory post hoc analyses to investigate its relationship with the COMT genotype. This analysis suggested that FA only affected COMT Met carriers who had been randomized to lamotrigine: there was an effect of FA randomization in the Met−lamotrigine group (*F*_1,114_=24.1; *P*=.000003; Figure 3) that was absent from all other groups (*F* values <5; *P* values >.58; Figure 3).

The effects of the MTHFR genotype were less prominent. There was an interaction between COMT and MTHFR (*F*_1,114_=10.1; *P*=.002), which resulted from opposing effects of COMT genotype dependent on MTHFR genotype across all groups (QIDS‐SR16 change was greater in COMT Met carriers than in Val/Val homozygotes in MTHFR C carriers [*F*_1,114_=4.2; *P*=.043], but smaller in TT homozygotes [*F*_1,114_=5.5; *P*=.020]). However, this did not interact with FA and/or lamotrigine randomization (*F* values <1; *P* values >.38). There were no other main or interactive effects (*F* values <3.9; *P* values >.05). There were no main or interactive effects involving FOLH genotype (*F* values <3.7; *P* values >.05).

### 3.3 FA supplementation increased folate and decreased total homocysteine

We explored whether biochemical indices of one‐carbon metabolism (either indices at baseline or their change over time) related to clinical outcome, as well as testing whether folate levels increased (and total homocysteine decreased) over time in those randomized to FA, as would be expected.

There were no differences in baseline biochemical measures (Table 1) between randomization groups (*F* values <1.8; *P* values >.16, except *F*_3,110_=2.3; *P*=.083 for total homocysteine; Table 1). There were no correlations between the primary clinical outcome and either the biochemical measures at baseline or changes in these measures from baseline to 12 weeks in the group as a whole (Spearman's rhos: −0.1 to +0.12; *P* values >.21). However, in those allocated FA, there was a correlation between the primary clinical outcome and baseline folate (Spearman's rho=−0.265; *P*=.039, i.e. those with higher baseline folate had a better clinical outcome at 12 weeks) but not the other biochemical variables examined (Spearman's rhos: −0.17 to 0.26; *P* values >.089).

**Figure 4:**
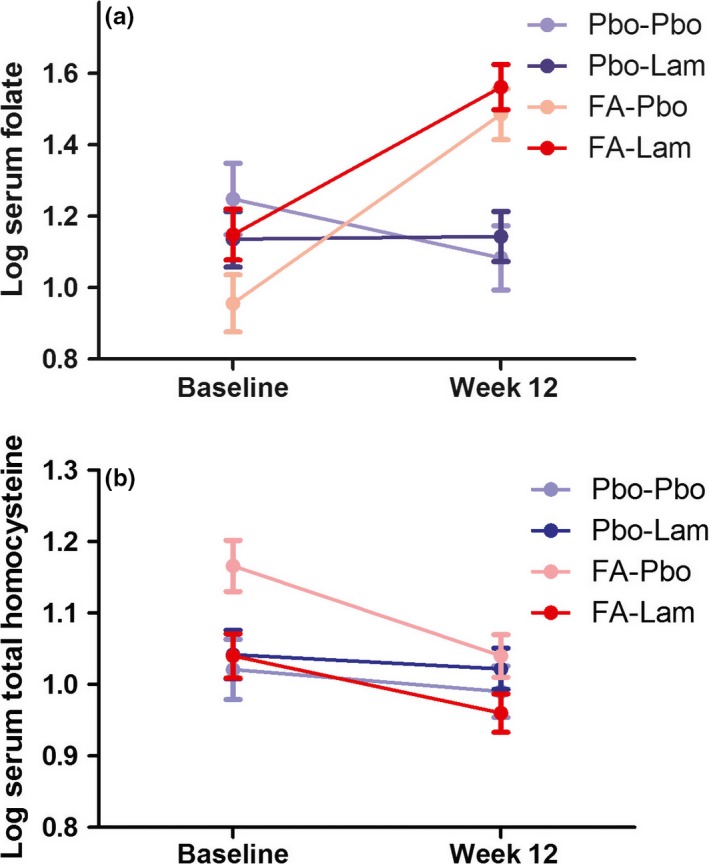
Folic acid (FA) administration robustly altered one‐carbon metabolism. Serum folate increased (A), and total homocysteine decreased (B), from baseline to 12 weeks in those randomized to FA (shown in red; n=43 for folate; n=39 for total homocysteine), but not placebo (shown in blue; n=32 for folate; n=29 for total homocysteine). Darker colours indicate those randomized to lamotrigine (Lam) and lighter colours those randomized to placebo (Pbo)

Folate levels increased from baseline to 12 weeks in those randomized to FA (Figure 4). Thus, there was an interaction between FA and time (*F*_1,67_=46.2; *P*=4×10^−8^), due to an increase from baseline in the FA (*F*_1,67_=78.0; *P*=7.6×10^−13^) but not the FA placebo group (*F*_1,67_=1.8; *P*=.19). Thus, folate levels differed between those randomized to FA and FA placebo at 12 weeks (*F*_1,67_=31.9; *P*=3.5×10^−7^) but not at baseline (*F*_1,67_=1.9; *P*=.17). There were also main effects of FA (*F*_1,67_=4.9; *P*=.03) and time (*F*_1,67_=22.8; *P*=.00001). However, there was no main effect of FOLH1 group (*F*_1,67_=0.5; *P*=.49) or lamotrigine (*F*_1,67_=0.3; *P*=.61), nor any other interactive effects (*F* values <3.3; *P* values >.075). Consistent with these findings, there was no main effect of FOLH1 group on baseline folate (*F*_1,114_=0.1; *P*=.75) when all participants (including those lacking 12‐week biochemistry data) were included in the analysis.

Homocysteine levels decreased from baseline to 12 weeks in those randomized to FA (Figure 4). There was a main effect of time (*F*_1,62_=11.8; *P*=.001), and a time‐by‐FA interaction (*F*_1.62_=9.2; *P*=.004), due to a decrease in homocysteine from baseline to 12 weeks in those randomized to FA (*F*_1,62_=22.8; *P*=.00001) but not FA placebo (*F*_1.62_=0.07; *P*=.79). There was also a main effect of COMT (*F*_1,62_=4.8; *P*=.032; Met carrier>Val/Val), as well as a lamotrigine−FA interaction (*F*_1,62_=5.5; *P*=.022) that appeared to result from the numerical differences in baseline homocysteine levels between randomization groups (Table 1). There were no other main or interactive effects (*F* values <2.2; *P* values >.14). Consistent with these findings, there was no main effect of COMT on baseline total homocysteine (*F*_1,111_=1.3; *P*=.26) when all participants (including those lacking 12‐week biochemistry data) were included in the analysis.

There were no main or interactive effects of time or randomization group on vitamin B_12_, total transcobalamin, or holo‐transcobalamin (*F* values <3.3; *P* values >.76), other than a main effect of time (*F*_1,71_=6.4; *P*=.014) on vitamin B_12_ levels (baseline>12 weeks).

A number of the biochemical measures were correlated with one another (Table 2).

**Table 2:** Intercorrelations between biochemical baseline values and week 12−baseline change scores

		Baseline homocysteine (log_10_)	Baseline vitamin B_12_ (log_10_)	Baseline Holo‐TC (log_10_)	Baseline total TC (log_10_)	Folate change (week 12−baseline; log_10_)	Homocysteine change (week 12−baseline; log_10_)	Vitamin B_12_ change (week 12−baseline; log_10_)	Holo‐TC change (week 12−baseline; log_10_)	Total TC change (week 12−baseline; log_10_)
Baseline folate (log_10_)	Spearman's rho	−**0.408**	**0.350**	**0.394**	0.027	−**0.239**	**0.569**	−0.136	−0.160	−0.117
	*P*	**.0000067**	**.0001166**	**.000013**	.772	**.039**	**.00000013**	.246	.174	.318
	N	**114**	**116**	**115**	116	**75**	**74**	75	74	75
Baseline homocysteine (log_10_)	Spearman's rho		−**0.318**	−**0.343**	0.002	0.203	**−0.621**	**0.277**	**0.238**	0.069
	*P*		**.00012**	**.0006**	.983	.086	**.0000000035**	**.018**	**.042**	.563
	N		**114**	**114**	114	73	**74**	**73**	**73**	73
Baseline vitamin B_12_ (log_10_)	Spearman's rho			**0.751**	0.160	−0.133	0.170	−**0.570**	−0.183	−0.132
	*P*			**3.9×10** ^**−22**^	.087	.255	.147	**.00000010**	.118	.258
	N			**115**	116	75	74	**75**	74	75
Baseline Holo‐TC (log_10_)	Spearman's rho				0.317	−0.235	0.232	−**0.462**	−**0.363**	−0.159
	*P*				.001	.043	.046	**.000034**	**.001**	.177
	N				115	74	74	**74**	**74**	74
Baseline total TC (log_10_)	Spearman's rho					−0.020	−0.038	−0.116	−0.146	−**0.574**
	*P*					.865	.745	.321	.216	**.000000072**
	N					75	74	75	74	**75**
Folate change (week 12−baseline; log_10_)	Spearman's rho						−**0.415**	0.195	0.205	0.027
	*P*						**.00027**	.094	.079	.820
	N						**73**	75	74	75
Homocysteine change (week 12−baseline; log_10_)	Spearman's rho							−0.034	−0.062	−0.050
	*P*							.773	.601	.672
	N							73	73	73
Vitamin B_12_ change (week 12−baseline; log_10_)	Spearman's rho								**0.519**	0.145
	*P*								**.0000022**	.216
	N								**74**	75
Holo‐TC change (week 12−baseline; log_10_)	Spearman's rho									0.164
	*P*									.164
	N									74

Nominally significant correlations are indicated in bold.

## 4 DISCUSSION

Our findings clarify and extend the primary analysis of CEQUEL in several ways. Firstly, they argue against a pharmacokinetic explanation for the observed clinical lamotrigine−FA interaction, since serum lamotrigine levels did not differ between those who were randomized to FA and those who were not. Secondly, the lack of a main effect of lamotrigine on depressive symptoms at 12 weeks appeared to be due to low or absent lamotrigine levels in some participants. Thirdly, we demonstrate that the interactive effect of FA and lamotrigine on clinical outcome may be modulated by the COMT genotype. These analyses highlight the value of biochemical and genetic measures to help explain and understand clinical trial outcomes.

### 4.1 Lamotrigine levels and clinical response

A notable aspect of our current findings is that a proportion of participants randomized to lamotrigine had low or undetectable serum levels. Critically, when we excluded these individuals there was a main effect of lamotrigine on the primary outcome, despite a reduction in sample size. This finding is in contrast to the primary intention‐to‐treat analysis of CEQUEL,^4^ in which there was no main effect of lamotrigine at 12 weeks (although its effect became significant at later time points). Thus, this analysis suggests that low serum lamotrigine levels in some individuals may have led to an underestimation of the efficacy of lamotrigine. We do not know whether the low or undetectable serum levels in some individuals randomized to lamotrigine simply reflect poor adherence; given the long half‐life,^21^ such individuals may have omitted more than just the dose on the day of testing. Our findings emphasize the importance of consistent adherence to prescribed medication for patients to benefit from lamotrigine and suggest that therapeutic drug monitoring should be more widely used in routine clinical practice as well as in all clinical trials of the drug.^22^

Although the low serum lamotrigine levels in a proportion of individuals led to an underestimate of the main effect of lamotrigine in the primary analysis of CEQUEL (Geddes et al., 2016), it does not explain the observed lamotrigine−FA interaction. Thus, although the interaction term no longer reaches significance when those with low or absent serum lamotrigine are excluded, exploratory post hoc tests indicate that (as in our primary analysis) the beneficial effect of lamotrigine remains confined to those randomized to FA placebo, and is absent in those randomized to FA.

A therapeutic range of 3‐14 mg/L is a widely used laboratory standard for lamotrigine, based on its use for epilepsy.^23^ Whilst consensus guidelines suggest the same therapeutic range for psychiatric indications,^20^ this is based on limited information. Naturalistic studies indicate that lamotrigine levels in psychiatric patients regularly lie below 3 mg/L,^22^^,^ 24, 25 leading some to suggest that a lower therapeutic range may be sufficient for bipolar disorder.^24^ However, prospective studies investigating how lamotrigine levels relate to changes in psychiatric symptoms are notably sparse and have focused on small, specialized patient groups. A plasma lamotrigine concentration of >3.25 mg/L was predictive of clinical improvement in a cohort of 38 patients with treatment‐resistant depression,^26^ whilst a small study of patients with rapid cycling bipolar disorder suggested that levels of 5 mg/L might be optimal.^27^ To our knowledge, our data represent the largest prospective study of the relationship between lamotrigine levels and clinical symptoms in bipolar disorder. Although caution should be exercised in generalizing our findings, given that all participants were also taking quetiapine, they suggest little direct relationship between lamotrigine levels and clinical symptoms. On the one hand, the results indicate that lamotrigine is clinically beneficial in bipolar depression when serum levels are near the lower end of its currently recommended therapeutic range. On the other hand, since all our participants showed levels well below the upper range of the therapeutic window, there is scope (at least with routine therapeutic monitoring) to increase the lamotrigine dose considerably beyond the 200 mg given here, with the potential for greater efficacy.

### 4.2 One‐carbon metabolism and clinical response

We explored a number of biochemical measures of one‐carbon metabolism in order to investigate the possible biological basis of the lamotrigine−FA interaction observed in our primary analysis. In the event, none shed light on this question (for example, clinical improvement did not correlate with serum folate or total homocysteine levels, although it remains possible that correlations may have been observed with other markers of one‐carbon metabolism, e.g. 5‐methyltetrahydrofolate^5^). However, the biochemical data confirmed that FA augmentation robustly both increased folate and decreased total homocysteine, and these changes were negatively correlated, in line with expectations (Figure 1).

We also studied the relationship between functional polymorphisms in genes related to one‐carbon metabolism and clinical symptoms. Whilst FOLH1 and MTHFR had little effect, COMT genotype modulated the effect of FA. Specifically, the negative impact of FA on lamotrigine response was only seen in COMT Met carriers (Figure 1). Clearly this finding, like the lamotrigine−FA interaction itself, requires confirmation since sample sizes for each genotype‐defined subgroup are very small. However, if true, it may provide clues as to the biological mechanisms underlying the lamotrigine−FA interaction. For example, given its place in the homocysteine cycle, the presence of the COMT Met allele should reduce the conversion of S‐adenosylmethionine to S‐adenosylhomocysteine in the presence of FA (which serves to drive the conversion of homocysteine to methionine). This in turn could plausibly reduce the amount of homocysteine converted into cystathionine and, ultimately, glutathione (Figure 1). Glutathione is the main antioxidant in the brain and lamotrigine's beneficial effect on oxidative stress appears to be mediated via increases in glutathione.^14^, ^15^ A range of model systems could be used to investigate the relationship between lamotrigine, FA and glutathione, and how this might be altered by changes in COMT activity. If results prove promising, these relationships could then be explored further in patient populations.

## 5 CONCLUSIONS

Our secondary analyses help clarify the findings from the CEQUEL trial and have implications for future studies of lamotrigine and FA. First, they suggest that the lack of a main effect of lamotrigine on the primary outcome (change in depressive symptoms from baseline to 12 weeks) may be due in part to low lamotrigine levels in some participants. Therefore, where possible, we recommend that future randomized controlled trials include therapeutic drug monitoring in their protocols. Second, the negative lamotrigine−FA interaction observed in the primary analysis is not explained by a simple pharmacokinetic effect, nor mediated by the measures of one‐carbon metabolism studied here. Whilst the unexpected nature of the lamotrigine−FA interaction means that it cannot be accepted as fact, its potential importance means that it should not simply be dismissed as a false positive. Given the cost associated with replicating this finding directly, we believe it may be more appropriate to conduct mechanistic studies to assess whether there is a plausible biological basis of the interaction. Our findings, suggesting that COMT activity (as proxied by COMT Val^158^Met genotype) modulates the observed interaction, provide a starting point for future hypothesis‐driven studies. Finally, the results show the value of measuring biochemical and genetic indices, along with other putative biomarkers, to enhance the value of clinical trials in this field.
